# Improving Embryonic Stem Cell Expansion through the Combination of Perfusion and Bioprocess Model Design

**DOI:** 10.1371/journal.pone.0081728

**Published:** 2013-12-10

**Authors:** David Yeo, Alexandros Kiparissides, Jae Min Cha, Cristobal Aguilar-Gallardo, Julia M. Polak, Elefterios Tsiridis, Efstratios N. Pistikopoulos, Athanasios Mantalaris

**Affiliations:** 1 Department of Surgery & Cancer, Imperial College London, London, United Kingdom; 2 Department of Chemical Engineering, Imperial College London, London, United Kingdom; Baylor College of Medicine, United States of America

## Abstract

**Background:**

High proliferative and differentiation capacity renders embryonic stem cells (ESCs) a promising cell source for tissue engineering and cell-based therapies. Harnessing their potential, however, requires well-designed, efficient and reproducible expansion and differentiation protocols as well as avoiding hazardous by-products, such as teratoma formation. Traditional, standard culture methodologies are fragmented and limited in their fed-batch feeding strategies that afford a sub-optimal environment for cellular metabolism. Herein, we investigate the impact of metabolic stress as a result of inefficient feeding utilizing a novel perfusion bioreactor and a mathematical model to achieve bioprocess improvement.

**Methodology/Principal Findings:**

To characterize nutritional requirements, the expansion of undifferentiated murine ESCs (mESCs) encapsulated in hydrogels was performed in batch and perfusion cultures using bioreactors. Despite sufficient nutrient and growth factor provision, the accumulation of inhibitory metabolites resulted in the unscheduled differentiation of mESCs and a decline in their cell numbers in the batch cultures. In contrast, perfusion cultures maintained metabolite concentration below toxic levels, resulting in the robust expansion (>16-fold) of high quality ‘naïve’ mESCs within 4 days. A multi-scale mathematical model describing population segregated growth kinetics, metabolism and the expression of selected pluripotency (‘stemness’) genes was implemented to maximize information from available experimental data. A global sensitivity analysis (GSA) was employed that identified significant (6/29) model parameters and enabled model validation. Predicting the preferential propagation of undifferentiated ESCs in perfusion culture conditions demonstrates synchrony between theory and experiment.

**Conclusions/Significance:**

The limitations of batch culture highlight the importance of cellular metabolism in maintaining pluripotency, which necessitates the design of suitable ESC bioprocesses. We propose a novel investigational framework that integrates a novel perfusion culture platform (controlled metabolic conditions) with mathematical modeling (information maximization) to enhance ESC bioprocess productivity and facilitate bioprocess optimization.

## Introduction

Embryonic stem cells (ESCs) have the potential to self-renew limitlessly and differentiate into any somatic cell type, which make them a promising cell source for use in tissue engineering & regenerative medicine and drug discovery applications [1]. Such applications require bioprocessing methodologies that are efficient and cost-effective [2]. Current cell culture methodologies present a bottle-neck in ESC implementation by being inefficient and sub-optimal. For instance, ESC culture is known to be bioprocess-dependent, exemplified by unscheduled differentiation in agitated cultures [3] as well as maintaining teratoma-forming *Oct3/4^+^* cells following differentiation [4].

ESCs exist within an equilibrium of sub-populations between a ‘naïve’ state possessing full pluripotency capacity and ‘primed’ ESCs that are poised to differentiate [5]. This equilibrium is affected, by, among others, extrinsic cues including fibroblast growth factor (*FGF*) signaling [6], culture substrates [7], [8], oxygen [9], nutrient/metabolite content [10], [11] and pH [12], and kinase inhibitors [13]. Furthermore, it has been reported that early passage human ESCs (hESCs) adapted to ‘standard’ culture conditions exhibit differences in transcriptional profiles, growth and culture re-initiation [14]. Therefore, ESC behavior is highly susceptible to the culture environment.

Three-dimensional (3D) culture substrates such as micro-carriers [15], [16], polymeric scaffolds [10] and hydrogels [17], [18] offer several advantages in the culture of ESCs and their derivatives. These include recapitulation of 3D native *in vivo* structures [17] and support of prolonged ESC culture [18], [19]. Furthermore, 3D cultures facilitate high density cellular growth [15], [16]. Alas, such high density cultures generate intra-day nutrient gradients (in between daily feedings) [16] and produce metabolites such as lactate that surpass critical levels, which are detrimental to ESC pluripotency and proliferation [10]. Consequently, reducing such metabolic stresses has been shown to aid significant increases in total cell density [15].

To elucidate the issue of the metabolic status of ESCs and the influence of metabolic by-product accumulation over toxic levels on ESC pluripotency, a combined experimental/modeling platform has been developed that enables identification of limiting behavior and regulates metabolic well-being to enhance ESC self-renewal capacity. Mathematical models have gained relevance given the increasingly higher amount of available biological data since they facilitate gaining additional insight from existing data [20]. Whereas traditional batch cultures, which retain culture medium for the duration of the culture, lead to the accumulation of metabolites (such as lactate and ammonia) past inhibitory levels, perfusion cultures, in contrast, facilitate environmental homeostasis, diluting metabolites and maintaining sufficient levels of nutrients [21]. The results suggest a novel mechanism of how inhibitory levels of metabolites promote the propagation of a less potent ESC sub-type, whereas diluting the metabolic stress experienced, by keeping metabolite levels below critical levels favours ‘naïve’ ESC propagation. Despite limited knowledge of regulatory processes that connect the culture environment to intrinsic stem cell attributes, an unstructured model was developed based on the premise that exposure to metabolites over critical levels mediate changes in mRNA expression levels, thereby determining the proportion of different stem cell sub-populations. A good agreement between experiments and modeling could lead towards the model–based optimization of ESC bioprocesses.

## Materials and Methods

### ESC Sub-culture, Encapsulation and Bioreactor Culture

**Figure 1:**
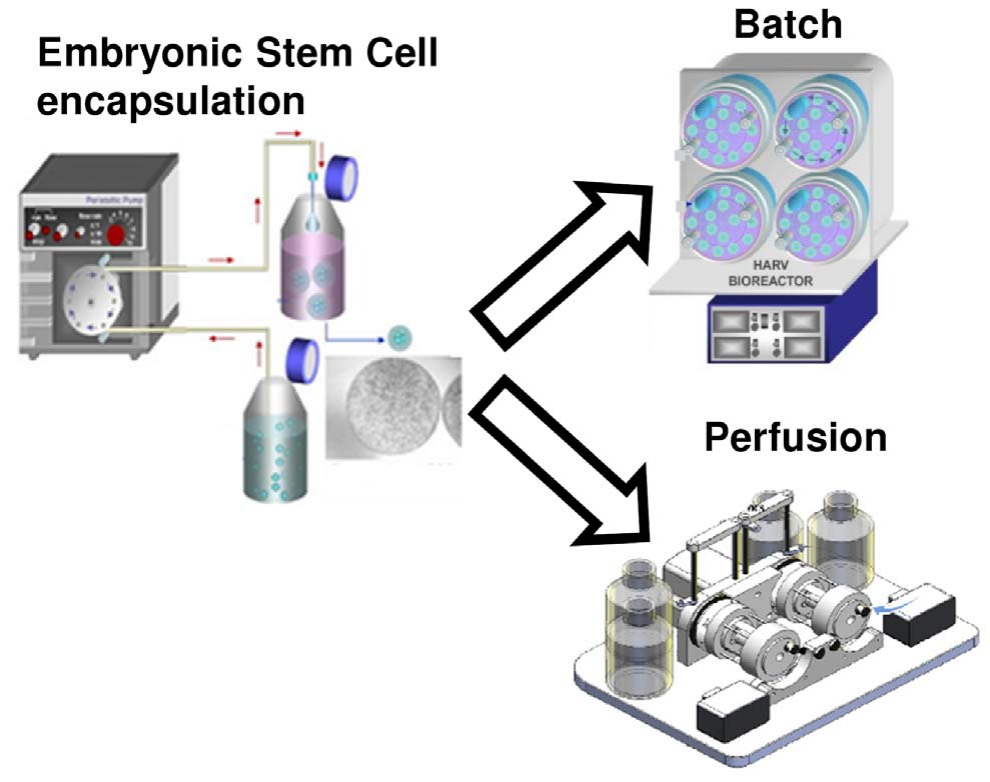
Schematic of the experimental design. mESCs were encapsulated in alginate hydrogels, as described previously [17], [22], and cultured within a batch operated HARV bioreactor and a custom-built perfusion bioreactor.

E14Tg2a mESCs (ATCC, UK) were cultured on T-75 tissue culture flasks pre-treated with 0.1% (w/v) gelatin (Sigma-Aldrich, Poole, UK), as previously reported [17], [22]. Encapsulation of single mESCs at 2.5×10^7^ cells per ml of alginate (1.1% v/v) with gelatin (0.1% v/v) at a final hydrogel bead density of 2×10^4^ cells per bead was accomplished as previously described [17], [22]. The culture medium consisted of high glucose Dulbecco’s Modified Eagle’s medium supplemented with 10% (v/v) fetal calf serum (FCS), 100 U/ml of penicillin and 100 µg/ml streptomycin, 2 mM L-glutamine (all from Invitrogen, Paisley, UK), 0.1 mM 2-mercaptoethanol (Sigma, UK) and 1000 U/ml Leukemia Inhibitory Factor (LIF; Millipore, Watford, UK). Batch cultures were performed in 55 ml high aspect ratio vessel (HARV) bioreactors (Cellon, Luxembourg). Perfusion cultures were performed in a novel perfusion rotating wall vessel bioreactor [23], [24], which consisted of a 60 ml vessel fabricated using a dual sided silicone-polytetrafluoroethylene (PTFE) gas permeable membrane (Specialty Silicone Products inc., NY, USA). Both the batch and perfusion cultures contained approximately 500 beads per vessel and each experiment was performed in triplicate. The bioreactors were placed in 20% oxygen and 5% CO_2_ conditions. The batch cultures were fed once at the beginning of the culture period which lasted for 8 days, whereas medium was continuously supplied at 2.29 ml/hr in perfusion culture conditions. The experimental scheme is illustrated in Figure 1. The biochemical characterization of cellular properties requires the release of mESCs from the alginate-gelatin beads. This was accomplished as previously described [15] in a sterile buffer containing 50 mM of tri-sodium citrate dehydrate (Fluka, UK). The released cells were then centrifuged to obtain a pellet, washed in PBS before snap freezing and storage in −86°C until analysis.

### Media Analysis

Nutrient and metabolite concentrations were obtained using the Bioprofile 400 Analyzer (Nova Biomedical, Flintshire, UK) from culture supernatant samples (1.5 ml) collected at the indicated time points. These samples were snap frozen and stored at −86°C and analyzed collectively.

### LIF Immunoassay

LIF concentration was quantified using a mouse leukemic inhibitory factor (mLIF) quantitative sandwich immunoassay (R&D systems, Abingdon, UK) according to the manufacturer’s instructions. Briefly, the assay utilizes a polyclonal antibody specific for mLIF pre-coated onto a 96 well format micro-plate, followed by the addition of culture supernatant (50 µl) at the indicated time points before incubating for 2 hours at room temperature. After washing off excess supernatant, a mLIF conjugate was added to the growth factors bound to the antibody coated micro-well surfaces. Substrate solution and stop solutions were added prior to reading on an absorbance micro-plate reader (Bio-Tek instruments Inc. Vermont, USA) at 450 nm with 570 nm correction. LIF concentration values were obtained from a standard curve generated from known quantities of mLIF provided by the manufacturer.

### Live/Dead Assay

Live/dead staining was conducted *in situ* for the 3D mESC alginate constructs, as descrived previously [25]. Briefly, alginate hydrogel beads were incubated at 37°C for 30 min in the dark using the live/dead cytotoxicity kit consisting of 4 mM ethdium homodimer-1 and 2 mM calcein AM solution (Invitrogen, Paisley, UK). Following staining, the 3D alginate mESC constructs were washed thoroughly with PBS and imaged within half an hour. The images were captured using an Olympus BX51 microscope and analysisD software (Olympus) without post-processing image manipulation.

### DNA Quantification

Total cell numbers were obtained indirectly from DNA content. A similar protocol to Randle et al [22] with minor modifications was used. Briefly, the digested cell supernatant was combined with PicoGreen® reagent (Invitrogen, Paisley, UK) as per the manufacturer’s instructions and analysed using an excitation/emission wavelength of 365 nm/460 nm in a MFX microtiter plate fluorometer (Dynex Technologies, West Sussex, UK). The normalized fluorescence readings were compared against a standard curve generated using known cell numbers.

### Quantitative PCR

Total RNA was extracted from the collected mESCs using the RNeasy kit (Qiagen, West Sussex, UK) according to the manufacturer’s instructions and quantified using a BioPhotometer plus (Eppendorf UK, Cambridge, UK). Relative gene expression was analyzed by conducting real-time quantitative polymerase chain reaction (qPCR) on the genes of interest. The SensiMIX™ SYBR No-ROX One-Step kit (Bioline, London, UK) was employed, which combines cDNA synthesis with PCR amplification within a single assay according to the manufacturer’s instructions. The One-Step kit is especially suitable when mRNA content is not limiting and high numbers of PCR reactions are required, reducing experimental time. Each PCR reaction consisted of 0.2 µM of primer (sense and anti-sense), 12 units of RNase inhibitor, 80 ng of total RNA and 1× SensiMix SYBR solution containing SYBR® green I dye dNTPs, reverse transcriptase, hot-start DNA polymerase, SensiTaq and 3 mM MgCl. Relative gene expression analysis was conducted using the 2^−ΔΔCT^ method [26] to calculate the relative fold differences between the normalizing value (T = 0, beginning of experiment) and glyceraldehyde 3-phosphate dehydrogenase (GAPDH) as the reference gene. Primer details are summarized in Figure S1.

### Statistical Analysis

Statistical analysis was carried out using one-way analysis of variance (ANOVA; Dunn’s method) for analysis consisting of more than 2 groups using SigmaStat 3.5 software (Scientific solutions SA, Pully-Lausanne, Switzerland). For analysis consisting of only 2 groups, a student’s *t*-test was applied (Microsoft, UK). Statistical significance was deemed at p<0.05.

### Mathematical Model

An unstructured mathematical model describing cell growth, metabolism and expression of selected genes was developed as presented below. In an attempt to overcome the lack of mechanistic knowledge regarding the precise effects of metabolic stress on the potency of ESCs, the presence of only 2 populations in the culture with distinct growth kinetics was assumed as an approximation of reality where multiple populations possibly exist. Specifically, *X_U_* refers to undifferentiated ESCs (‘naïve’ ESCs) while cells of a lower ‘potency’ are lumped under the term ‘differentiated’ *X_D_* (‘primed’ ESCs). Based on the approximate ratio of ‘naïve’ to ‘primed’ ESCs in similar culture conditions being reported to be 9∶1 [5], an estimate of 85% *X_U_* type and 15% *X_D_* type cells as the initial condition was employed. Despite the fact that the model accounts for two distinct populations of cells, nutrient uptake and metabolite production is averaged over the total of viable cells (*X_V_*). Gene expression of 3 genes (*Rex1*, *Dppa3* and *Fgf5*) has been modeled based on the assumption that the toxicity of accumulating metabolites can affect the expression of genes associated with pluripotency and/or differentiation [10].

A material balance for viable undifferentiated and differentiated cells within the bioreactor is given by the following equations:
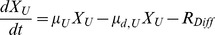
(1)

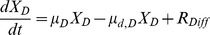
(2)where *X_U_* and *X_D_* (cells bead^−1^) are the concentrations of viable undifferentiated and differentiated cells in the bioreactor, respectively. *R_Diff_ (h^−1^)* is the rate of transfer of cells between the two cell populations while μ and µ_d_ are the specific growth and death rates (h^−1^), respectively. Total viable cell concentration *X_v_* is given by the sum *X_D_* and *X_U_*

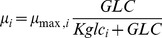
(3)where *µ_max,i_* (h^−1^) is the maximum theoretical growth rate for cells of type (i). (i) denotes either undifferentiated (U) or differentiated (D) cells. *K_glci_* (mM) is the Monod constant for the primary nutrient, glucose.
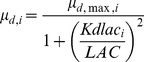
(4)
*µ_d,max,i_* represents the maximum specific death rate (h^−1^) and *K_dlaci_* describes the rate of cell death by lactate.

The expression of *Rex1* varies significantly in mESCs between their ‘naïve’ and ‘primed’ states; therefore, *R_Diff_* which is the rate of cell transfer between the 2 populations is given as a function of the relative expression levels of *Rex1*.
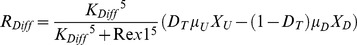
(5)where



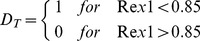
(6)D_T_ is a binary variable which determines the direction of cell transfer between the two populations (comparable to reversibility of ‘naïve’ and ‘primed’ ESC states [27]) depending on the relative expression level of *Rex1*.

The material balance for glucose is given by:
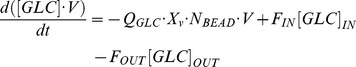
(7)with,
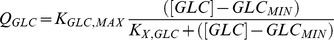
(8)where *K_GLC,MAX_* (mM cell−1 h−1) and *K_X,GLC_* (mM) denote the maximum uptake rate of glucose by the cells and the Monod constant for glucose consumption, respectively. *N_BEAD_* refers to the number of beads in the bioreactor. Similarly, glutamine concentration is given by:
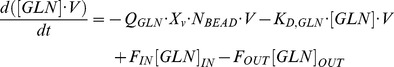
(9)with,
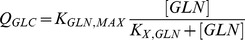
(10)where *K_GLN,MAX_* (mM cell−1 h−1) and *K_X,GLN_* (mM) denote the maximum uptake rate of glutamine by the cells and the Monod constant for glutamine consumption, respectively. The only difference is the term containing glutamine degradation, which has been shown to degrade spontaneously in mammalian culture conditions [28]. Similarly, mass balances can be formulated to describe the temporal evolution of the concentrations of the primary by-products of cell metabolism. More specifically, for lactate:
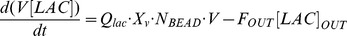
(11)with,



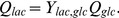
(12)Similarly for ammonia:
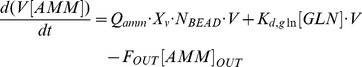
(13)with,
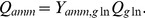
(14)
*Q_lac_* and *Q_amm_*, represent the specific production rates (mmol cell−1 hr−1) while *Y_lac,glc_* and *Y_amm,gln_* represent the yield of the particular product on its primary nutrient (mM/mM).

Gene expression was modeled using Hill functions following the hypothesis that the accumulation of toxic metabolites reduces pluripotency of ESCs. Therefore, expression of ICM genes *Rex1*, *Dppa3* was inversely correlated with metabolic stress but positively correlated with the expression of the differentiation marker, the primitive ectoderm (PrEct) gene *Fgf5*. *Rex1* and *Dppa3* are strongly expressed in the inner cell mass (ICM) of the murine blastocyst, but are significantly reduced in expression during the PrEct stage of development [29]. Gene expression of ‘naïve’ and ‘primed’ ESC enriched sub-populations were also found to differ appreciably [5], [27]. ‘Primed’ sub-populations of ESCs were observed to have more than 10× higher expression of *Fgf5* compared to the ‘naive’ population [5] analogous to the up-regulation of *Fgf5* observed in the PrEct stage of development [30]. The considerable differences in expression between the ‘naïve’ and ‘primed’ ESC populations of these 3 genes justifies their inclusion in the model compared to genes such as *Oct3/4*, which have relatively uniform gene expression levels between ‘naïve’ and ‘primed’ ESCs [5]. Rex1 expression is given as:
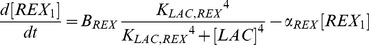
(15)


*B_REX_* (h^−1^) denotes the basal transcription rate, representing the transcription rate of the given gene at the assumed *X_U_*/*X_D_* ratio of 85∶15. *K_LAC,REX_* (mM) is the inhibition constant from lactate. *α_REX_* (h^−1^) is the mRNA degradation rate.

*Dppa3* expression is given as:
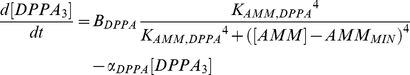
(17)


*B_DPPA_* (h^−1^) represents the basal transcription rate, while *K_AMM,DPPA_* (mM) is the inhibition constant from ammonia. *α_DPPA_* (h^−1^) is the mRNA degradation rate.

*Fgf5 e*xpression is given as:
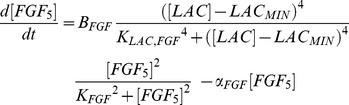
(16)


*B_FGF_* (h^−1^) represents the basal transcription rate, while *K_LAC,FGF_* (mM) is the inhibition constant from lactate. *Fgf5* gene transcriptional activity is reported to consist of 2 isoforms, full-length *Fgf5* mRNA and *Fgf5S* that expresses only partial genetic information [31]. It is assumed that the isoforms interact with each other thus *Fgf5* is modeled to regulate its own expression. *K_FGF_* is the Monod constant for *Fgf5* self-regulation and *α_FGF_* (h^−1^) is its degradation rate.

### Computational Methods

All parameter estimation, sensitivity analysis and model simulations were carried out on an Intel® Core^TM^2 Duo (E4600–2.4, 2.39) personal computer with 3.24 GB of RAM memory and implemented in the advanced process modeling environment gPROMS® (Process Systems Enterprise, 2009).

### Parameter Estimation and Global Sensitivity Analysis (GSA)

Experimental data from triplicate batch cultures of alginate encapsulated mESCs were used to estimate the model’s parameters. Table 1 summarizes all parameters contained in the model and their estimated values for batch operation. In order to account for the different metabolic characteristics of perfusion culture, some model parameters need to be adjusted. Instead of re-estimating the entirety of the model parameters, following the model development algorithm presented in [12], the parameter vector is partitioned into significant and insignificant parameters with the use of GSA. Parameters identified as insignificant retain the values estimated from the batch experiment while parameters identified as significant are re-estimated based on the results of the perfusion experiment and can be found in Table 2. More details of the GSA results can be found in Figure S2.

**Table 1:** Parameter values estimated from batch culture.

Parameter	Value	Units	Parameter	Value	Units
µ_max,U_	0.928	h^−1^	µ_max,D_	0.819	h^−1^
Kglc_U_	1	mM	Kglc_D_	10.813	mM
µ_d,max,U_	0.517	h^−1^	µ_d,max,D_	0.596	h^−1^
Kdlac_U_	0.212	mM	Kdlac_D_	1.618	mM
K_GLC,MAX_	5.285E-07	mM/cell	K_GLN,MAX_	8.330E-09	mM/cell
Kx_GLC_	45.924	mM	Kx_GLN_	9.893	mM
Y_LACGLC_	1.750	mM/mM	Y_AMMGLN_	0.814	mM/mM
GLC_MIN_	6.323	mM	K_Diff_	0.721	N/A
K_D,GLN_	0.146	h^−1^	b_REX1_	1.848	h^−1^
a_REX_	1.465	h^−1^	b_FGF_	2.724	h^−1^
α_FGF_	0.306	h^−1^	b_DPPA_	10	h^−1^
α_DPPA_	6.549	h^−1^	LAC_MIN_	4.020	mM
K_LAC,REX_	21.481	mM	K_FGF_	1.013	N/A
K_LAC,FGF_	14.539	mM	AMM_MIN_	1	mM
K_AMM,DPPA_	1.674	mM	–	–	–

Note: Refer to the main text for full details of abbreviations.

**Table 2:** Subset of significant parameter (identified by GSA) values re-estimated from perfusion culture.

Parameter	Value	Units	Parameter	Value	Units
µ_max,U_	13.693	h^−1^	K_GLN,MAX_	3.916E-08	mM/cell
Kglc_U_	141.901	mM	Y_LACGLC_	1.357	mM/mM
µ_d,max,U_	1.221	h^−1^	Y_AMMGLN_	1.769	mM/mM

## Results

### Batch Cultures Produce a Metabolic Stress

**Figure 2:**
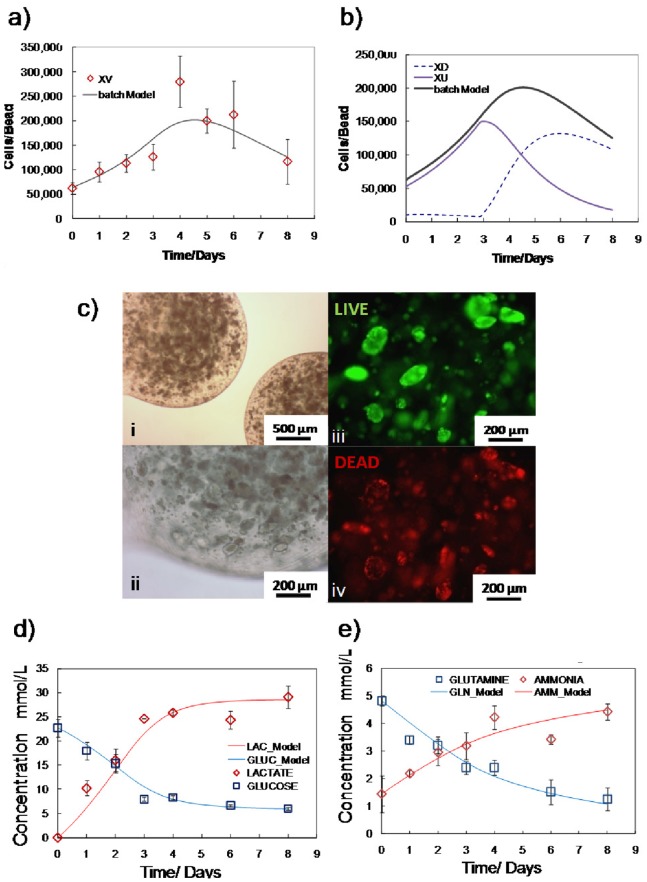
Batch culture growth kinetics, viability and metabolism. a) Total number of viable ESCs; experimental (◊) and model predictions (–); b) Simulation results of the different ‘naïve’ (*X_U_*) and ‘primed’ (*X_D_*) mESC sub-populations; c) Micrographs of the mESCs encapsulated in the alginate hydrogels at day 9 (i, ii); Live (green)/dead (red) fluorescence micrographs showing live cells (iii) forming colonies of <200 µm in diameter as well as a high proportion of dead cells (iv) at day 9; d) experimental (symbols) and simulation (lines) glucose and lactate concentration profiles; e) experimental (symbols) and simulation (lines) glutamine and ammonia concentration profiles. Experimental values represent mean±SD, N = 3.

The growth kinetics obtained in the (HARV) batch cultures demonstrated the typical lag, exponential, stationary and decline phases observed in batch cultures, as seen in Figure 2a. A peak in cell density is attained at 280±52×10^3^ cells/bead (day 4) followed by a steady decrease in cell numbers. Maximum specific growth rate for undifferentiated cells, µ_Xu_batch_max_, was estimated at 0.49 day^−1^ corresponding to a doubling time of 1.42 days. The mathematical model captures the kinetics of batch culture well with the apparent discrepancy of the peak cell density at end of the exponential phase at day 4. This under-prediction could be due to different growth characteristics of the differentiated population (*X_D_*), which experiences a sudden increase, not taken into consideration. Figure 2b shows the predicted concentrations of *X_U_* and *X_D_* cells after an assumed initial composition of 85% *X_U_* and 15% *X_D_*. During the early stages of the batch culture, a slight increase in the *X_U_*/*X_D_* ratio can be observed presumably due to the low concentrations of toxic metabolites and the availability of all nutrients in the media. At day 3, the model predicts a sharp transition of cells from the *X_U_* to the *X_D_* state as a result of surpassing toxic metabolite levels (lactate). In reality, the decrease in *X_U_* cells is expected to be more gradual, however, the model accounts only for two discrete cell states (*X_U_* and *X_D_*), whereas a greater number of distinct sub-populations is anticipated in reality. Cell viability was recorded between 90–95% throughout the culture period until day 8 whereby the viability dropped to ∼70% (data not shown). Live/dead assay confirmed these findings with representative images of cell colonies approximately 200 µm in diameter having a high proportion of dead cells (Figure 2c). The nutrient and metabolite content of the batch cultures was examined; specifically, glucose, glutamine (nutrients), lactate and ammonia (metabolites) were analyzed. A sharp decrease in glucose consumption was observed from day 3 onwards (Figure 2d) despite an abundance in glucose concentration at over 5 mmol/L. This drastic change in glucose metabolism may be attributed to the different metabolic requirements of the differentiated sub-population, or to the shortage of other essential nutrients, such as amino acids. The reduction in glucose consumption is concomitant, as expected, to the plateau in lactate accumulation (Figure 2d). Contrary to glucose metabolism, glutamine consumption continues throughout the culture period (Figure 2e). This could partially be attributed to the spontaneous degradation of glutamine in aqueous solutions at temperatures above 30°C [28]. It is worth noting that by day 2, lactate concentration was at 16 mM, which is a level previously shown to inhibit mESC propagation and pluripotency [10]. Ammonia accumulation remained below inhibitory concentrations (4 mM) [32] until day 4. The model captured the temporal profiles of all nutrients and metabolites with no major discrepancies (Figure 2d, e).

**Figure 3:**
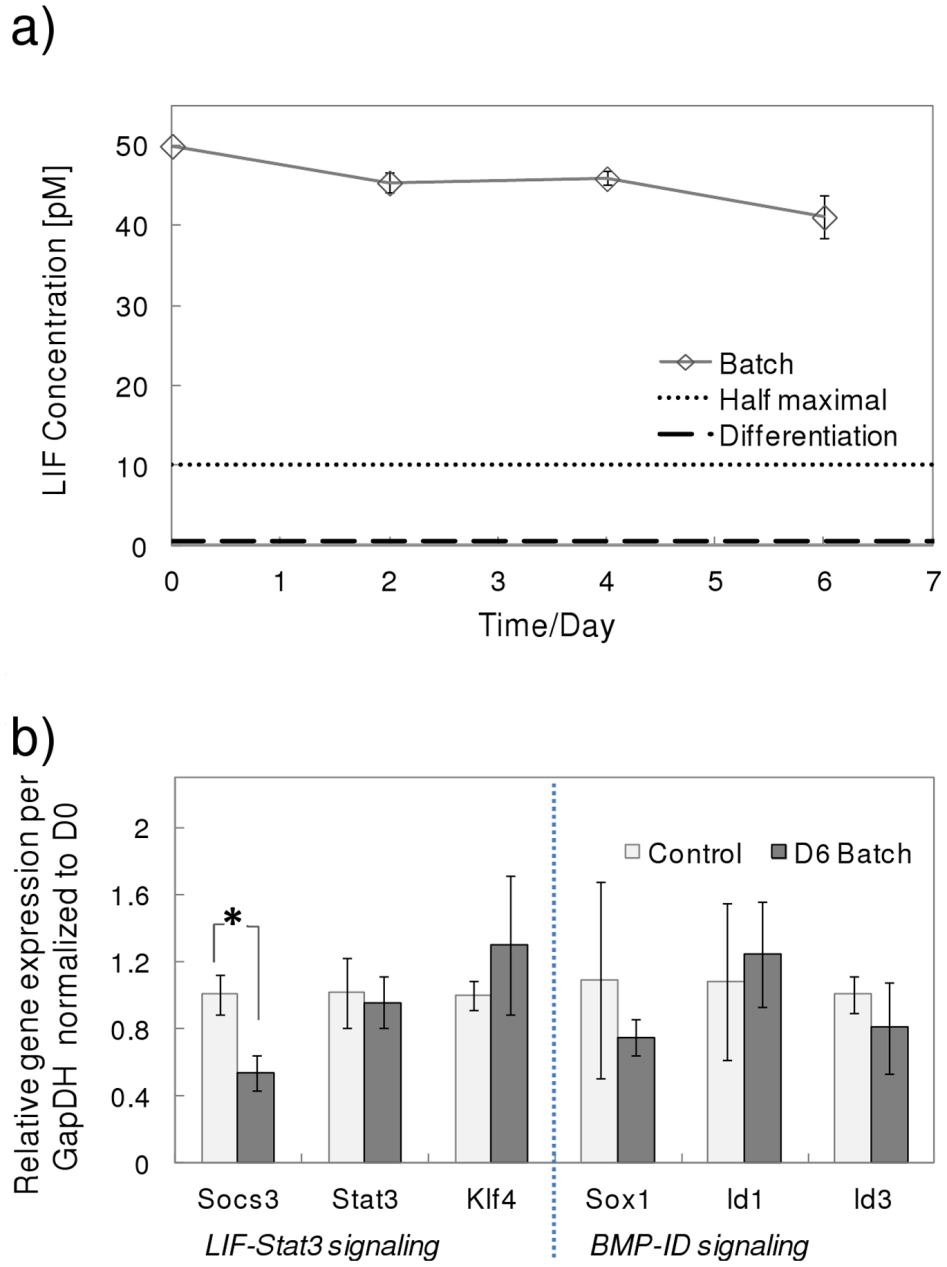
LIF concentration and associated gene expression in batch cultures. a) LIF growth concentration levels over the 6 day culture period remain significantly higher than the half maximal and differentiation threshold levels. b) Gene expression levels of *LIF-Stat3* signalling (*Socs3*, *Stat3*, *Klf4*) and *BMP-ID* signalling (*Sox1*, *Id1* and *Id3*) for control (day 0) and day 6 batch culture values. *P<0.05, Student t-test. Experimental values represent mean±SD, N = 3.

The ability of mESCs to self-renew in their pluripotent state is based on the elimination of pro-differentiation signals. One strategy to propagate undifferentiated mESCs is the provision of LIF and bone morphogenetic protein 2/4 (BMP) [33], which block mesoderm/endoderm and neuroectoderm differentiation, respectively. LIF stimulates the *Jak/Stat3* (Janus kinase/signal transducer and activator of transcription-3) pathway by phosphorylating *Stat3* (*pStat3*). Thereafter, *pStat3* initiates gene transcription [34], which is further regulated by *Socs3* (suppressor of cytokine signaling) [35] and associates with the pluripotency network via *Klf4* [36]. The LIF concentration was quantified in the batch cultures to ascertain whether it was depleted. It was observed that approximately 20% of the initial concentration of LIF was consumed by day 6 (Figure 3a), with over 40 pM LIF remaining in the supernatant throughout the batch culture. It has been reported that a concentration of 10 pM LIF maintains mESC self-renewal at half the maximum rate [37], whereas mESCs differentiate [38] at 0.5 pM of LIF. Consequently, LIF levels in the batch cultures remained abundant. Gene expression analysis of expression levels of *Stat3*, *Socs3* and *Klf4*, (transcription factors involved in LIF signaling) was examined. Figure 3b shows that *Stat3* and *Klf4* expression on day 6 was at similar levels to control values (day 0); however, *Socs3* levels were significantly lower. *Socs3* is a direct target of the LIF signaling pathway and has been shown to experience a more rapid and severe down-regulation compared to *Stat3* [36], [39] at lower LIF concentration levels. The down-regulation of *Socs3* expression suggests that LIF signaling may have been attenuated even in the presence of sufficient LIF. Maintenance of the pluripotent state also involves *BMP* factors signaling through *Id* (inhibitor of differentiation) proteins to impede neuroectoderm differentiation. Since the induction of *Id* expression can be stimulated by serum, *BMP2* or *BMP4*, growth factor signaling was indirectly evaluated through gene expression analysis of *Id1*, *Id3* and *Sox1* (a neuroectoderm differentiation gene). No significant variations in the expression of any of the genes between day 6 and the control group (day 0) was observed (Figure 3b), suggesting that growth factor supply was adequate to enable *BMP* signaling, that neutralized neuroectoderm differentiation signals.

**Figure 4:**
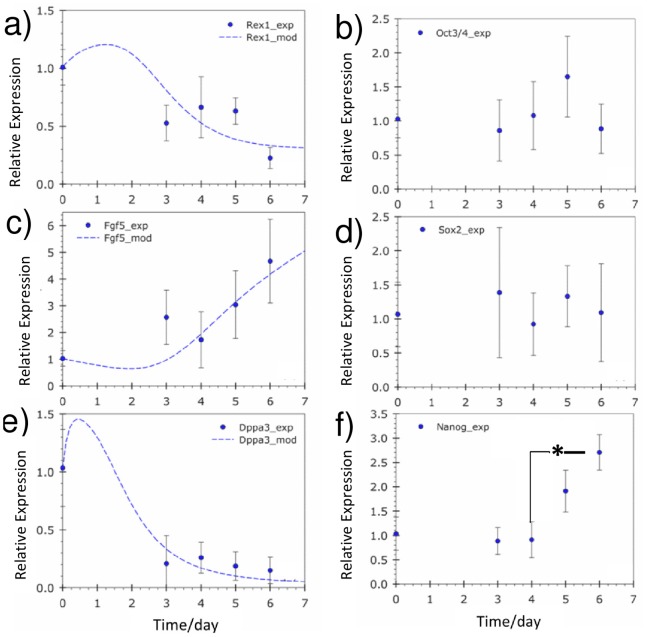
Pluripotency-related gene expression in batch cultures. Gene expression levels of a) *Rex1*, b) *Oct3/4*, c) *Fgf5*, d) *Sox2*, e) *Dppa3* and f) *Nanog*. Model simulation results are predicted for *Rex1*, *Fgf5* and *Dppa3* (line). *P<0.05, one-way ANOVA. Experimental values represent mean±SD, N = 3.

A panel of 6 genes was chosen to characterize pluripotency levels, as shown in Figure 4. The *Oct3/4*, *Sox2* and *Nanog* genes are essential to the pluripotency network [40]. Genes expressed in the pre-implantation ICM, *Rex1* [5] and *Dppa3* (Stella) [27], are known to be repressed in the epiblast concomitantly with an up-regulation of *Fgf5* [5]. Gene expression was normalized with both control values (day 0 cells) and expression of the house keeping gene glyceraldehyde 3-phosphate dehydrogenase (*GapDH*). Given that glucose consumption was reduced in the latter stages of the batch cultures and *GapDH* is an enzyme involved in glycolysis, its Ct (threshold crossing cycle number) values throughout the culture period were examined to ensure that housekeeping gene activity was not altered. The Ct values at the start of the batch cultures compared to the end of the batch cultures had a *p*-value higher than 0.18, signifying no statistical difference, affirming the stable expression levels of *GapDH* and its suitability as a housekeeping control. Nonetheless, *β-actin* and *18s* rRNA are other suitable housekeeping genes that can be used in place of *GapDH* to avoid potential changes due to altered metabolic activity. *Oct3/4* and *Sox2* gene expression was relatively stable throughout the batch cultures. Unexpectedly, an increase in *Nanog* expression was observed on day 6 in the batch cultures. This may be a result of attenuated LIF activity which reduces *Nanog*-repressing mitogen-activated protein kinase (MAPK) signals [36]. Alternatively, *Nanog* up-regulation could also be a result of the stressful metabolic conditions via the ‘parallel’ phosphatidylinositide 3-kinase (*Pi3K*) – *Akt* signaling [36]. The expression levels of *Rex1* and *Dppa3* decreased over culture time, whereas *Fgf5* expression increased. These results suggest that mESCs in batch cultures retained certain pluripotent characteristics but clearly lose ‘naivety’, becoming more lineage ‘primed’ compared to the initial population. The mathematical model captured the experimental trends successfully, both in terms of the decrease in *Rex1*, *Dppa3* and the increase in *Fgf5*. The increase in *Rex1* and *Dppa3* expression at the initial stages of the culture can be attributed to the ‘fresh’ and favorable culture environment. The experimental results confirm the hypothesis that accumulation of metabolites above toxic levels during batch cultures ‘primes’ mESCs for differentiation. The mathematical model was able to capture the experimental trends and predicted a shift in the composition of the initial population towards a higher percentage of differentiation ‘primed’ cells as a result of metabolic stress.

### Perfusion Removes the Metabolic Stress and Enhances Pluripotency

**Figure 5:**
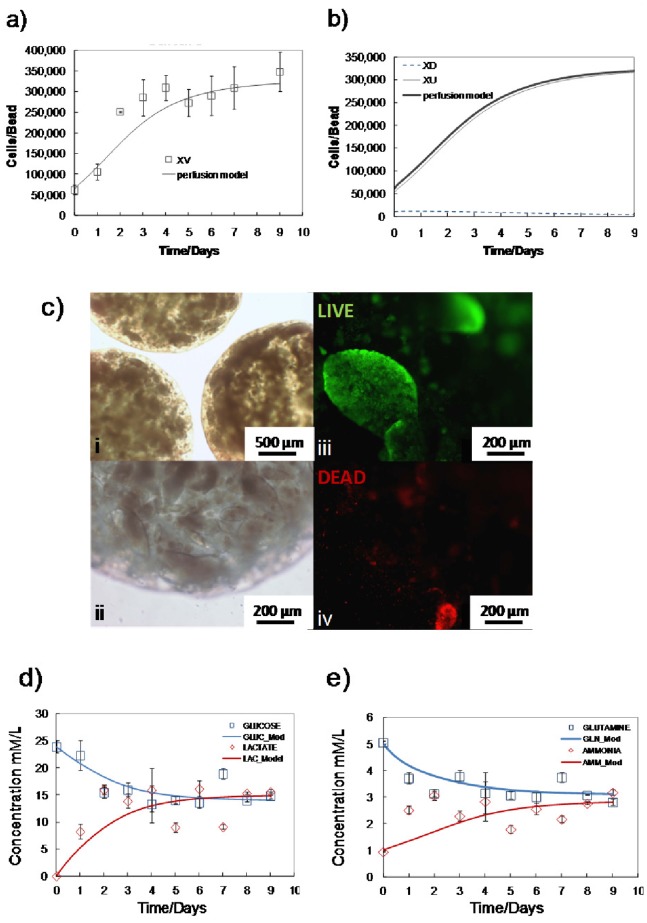
Perfusion culture growth kinetics, viability and metabolism. a) Total number of viable ESCs; experimental (□) and model predictions (–); b) Simulation results the cellular states of ‘naïve’ (*X_U_*) and ‘primed’ (*X_D_*) mESCs; c) Micrographs of the mESCs encapsulated in the alginate hydrogels at day 9 (i, ii); Live (green)/dead (red) fluorescence micrographs showing the live cells (iii) forming large colonies of 300–400 µm in diameter with minimal dead cells (iv) at day 9; d) experimental (symbols) and simulation (lines) glucose and lactate concentration profiles; e) experimental (symbols) and simulation (lines) glutamine and ammonia concentration profiles. Experimental values represent mean±SD, N = 3.

Perfusion facilitates the continuous dilution of metabolites and provision of fresh nutrients thus maintaining a relatively constant metabolic environment, which supports high density cellular growth [41]. Cell metabolism has been reported to depend on culture mode, whether batch or perfusion, in mammalian cells [42]. Consequently, the mathematical model needs to be adjusted to account for this difference in the mode of operation. The use of global sensitivity analysis (GSA) allows the partitioning of model parameters into significant and insignificant parameters, aiding the re-estimation of a mere subset of parameters with greater sensitivity (Table 2). Sensitivity indices have been shown to vary with culture time (as well as culture modality; i.e., batch versus perfusion) [43], therefore GSA was performed at 3 time points (2, 5 and 8 days, Figure S2) in an attempt to capture the various phases (lag, exponential growth and decline) of batch culture. A significant difference between batch and perfusion in the numerical values of *µ_max,U_* and *Kglc_U_* was observed. This is attributed to the gross approximation of the cellular composition to only 2 states with population-averaged values. A higher rate of cell expansion was observed during the early stages of perfusion in comparison to batch cultures. Cells reached a steady state density of 3.5×10^5^ cells per bead by day 3 (Figure 5a). This growth plateau was attributed to spatial constraints since metabolic activity did not significantly change. Perfusion supported a higher cell density compared to batch cultures; this cell density corresponds to approximately 3.2×10^6^ cells/ml which is comparable to the maximum cell density of other dynamic ESC micro-carrier cultures [15], [44]. The mathematical model was able to capture the experimental trends well albeit underestimating the rapid rate of cell growth observed during days 2–4 (Figure 5b). µ_Xv_perfusion_max_ was estimated to be 1.15 day^−1^ (0.60 day doubling time), which compares well with previous work on mESC micro-carrier expansion [44], [45]. Significantly, the model predicted that the perfusion cultures preferentially expand only the high quality undifferentiated (*X_U_*), which was in contrast to the batch cultures where 73% of cells at day 6 were predicted to be differentiated (‘culture primed’) mESCs (*X_D_*). Perfusion maintained adequate levels of nutrients while accumulation of metabolites remained below toxic levels (16 mM for lactate and 4 mM for ammonia). Consequently, the cells did not experience any unnecessary change in their metabolic activity. Viability remained high even though the hydrogel beads were packed with cell colonies, including larger-sized colonies (Figure 5c). The cell colonies were estimated to be in the range of 300–400 µm in diameter compared to 150–200 µm in batch cultures. In addition, the mathematical model captured the experimental results of nutrient/metabolite metabolism satisfactorily (Figures 5d, e). Differences in the metabolic activity between batch and perfusion cultures were observed, as expected. The Y_LACGLC_ was 1.36 for the perfusion cultures 1.75 for the batch cultures suggesting that a higher proportion of glucose enters the citric acid cycle (TCA) cycle. These results were further substantiated by the ammonia and glutamine metabolism data. Two possibilities emerge, that either cell metabolism was modified due to the mode of feeding or ESCs in different states of potency exhibit distinct metabolic characteristics.

**Figure 6:**
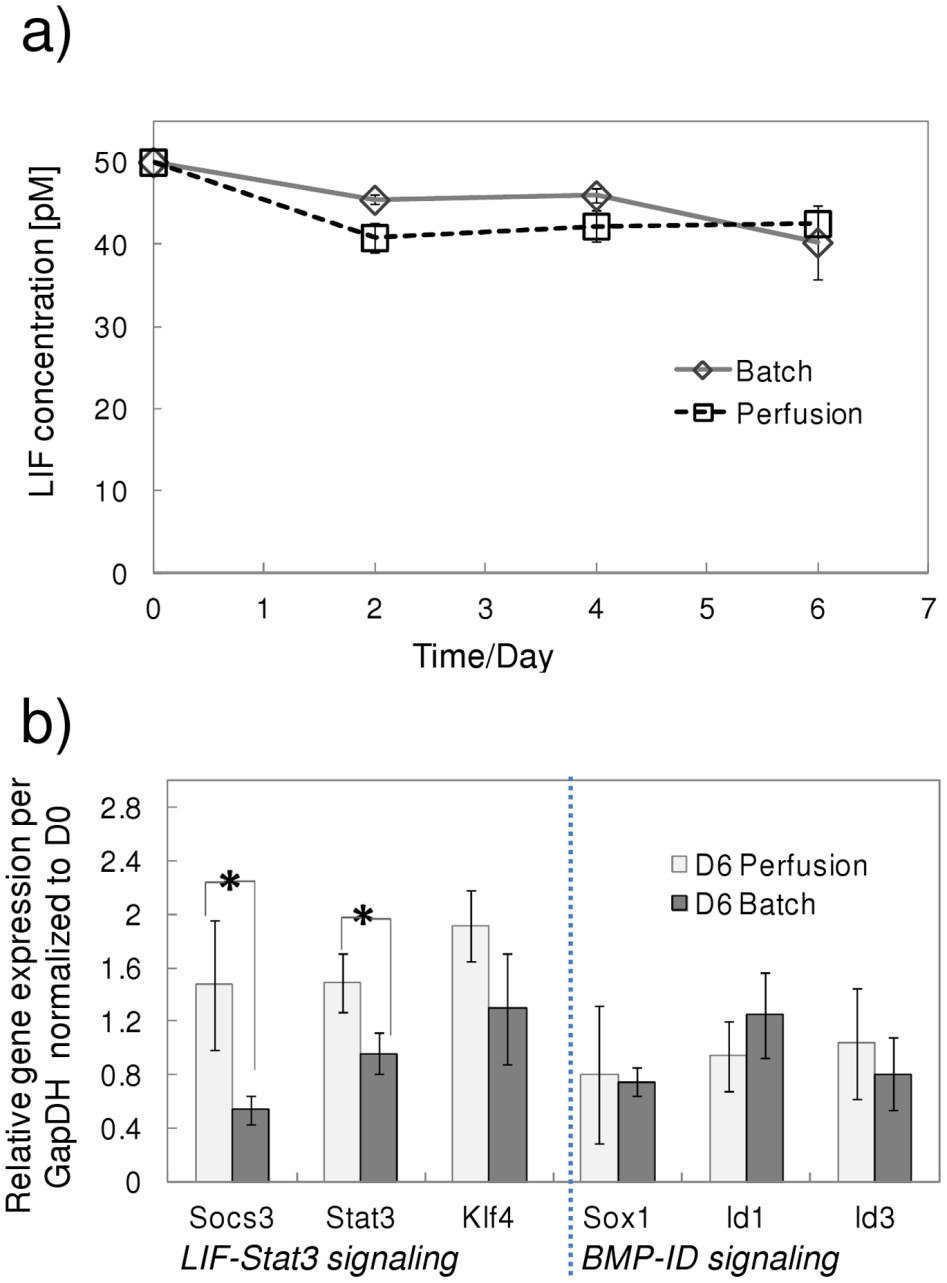
LIF concentration and associated gene expression in perfusion cultures. a) LIF growth concentration levels remain high in both the perfusion and batch cultures throughout the culture period. No difference in LIF concentration between the two operation modes was observed. b) Gene expression levels of *LIF-Stat3* signalling (*Socs3, Stat3, Klf4*) and BMP-ID signalling (*Sox1, Id1 and Id3*) for control (day 0) and day 6 batch culture values. *P<0.05, Student t-test. Experimental values represent mean±SD, N = 3.

A comparison of the LIF concentration in the perfusion and batch cultures shows that LIF levels were comparable throughout the culture period (Figure 6a). LIF reversibly binds to its receptor (*LIFR*) and is consumed primarily by receptor internalization and subsequent hydrolysis [46]. Under the assumption that a single LIF molecule binds with its receptor, estimates from literature values yield a LIF consumption rate of 2.2–7.4×10^9^ molecules per day at a cell concentration of 3×10^5^ cells per day [35], [47]. This value is 3 orders of magnitude lower than the experimentally measured LIF consumption rate. Such phenomena could be attributed to other sources of LIF consumption, internalization occurring at a higher rate, differences in ligand binding mechanism or any combination of the above. Given that LIF is reportedly stable for 7 days in culture, this theoretical analysis further substantiates the experimental findings that LIF supply was sufficient in both operating conditions. However, the expression of genes associated with LIF signaling (*Stat3* and *Socs3*) in the perfusion cultures was observed to be significantly higher than batch cultures (Figure 6b). This could be attributed to the higher levels of endogenously produced *gp130* ligands [48] at higher mESC densities. No significant differences were observed in the expression of *Sox1*, *Id1* and *Id3* signifying that neuroectoderm differentiation was held in check.

**Figure 7:**
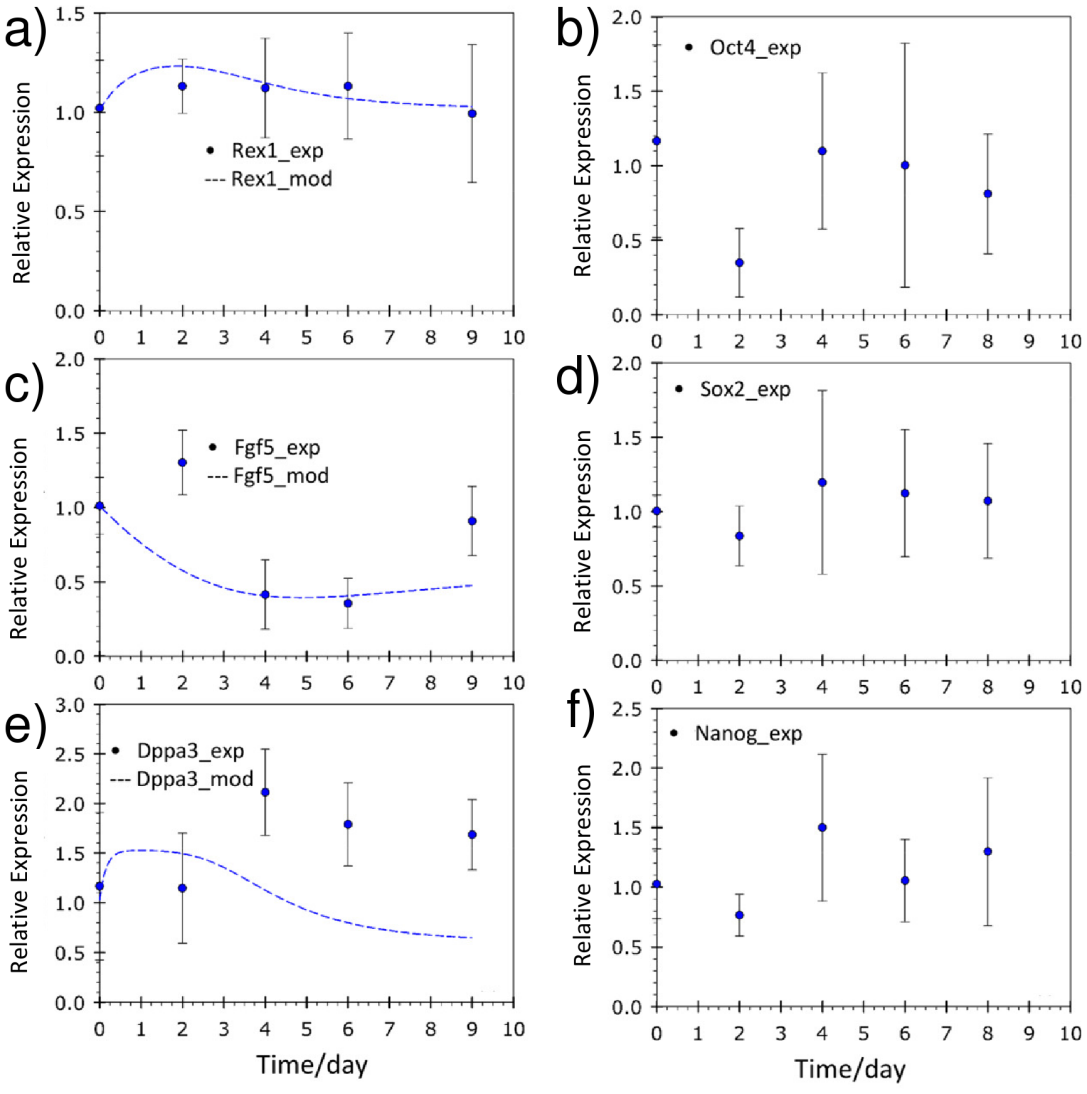
Pluripotency-related gene expression in perfusion cultures. Gene expression levels of a) *Rex1*, b) *Oct3/4*, c) *Fgf5*, d) *Sox2*, e) *Dppa3* and f) *Nanog*. Model simulation results are predicted for *Rex1*, *Fgf5* and *Dppa3* (line). *P<0.05, one-way ANOVA. Experimental values represent mean±SD, N = 3.

**Figure 8:**
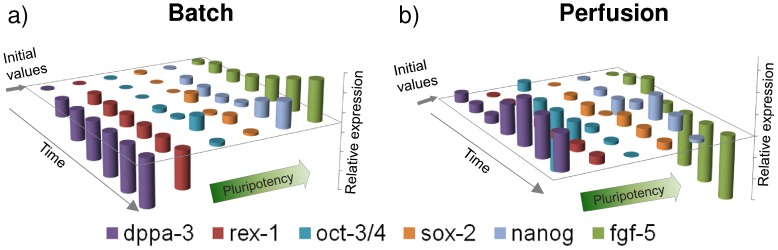
Gene expression differences between batch and perfusion cultures. The accumulation of metabolic stress within batch cultures (a) leads to the down-regulation of the expression levels of *Rex1* and *Dppa3* accompanied by the up-regulation of the *Fgf5*. Perfusion feeding (b) removes the metabolic stress resulting in the up-regulation of *Rex1* and *Dppa3* and the down-regulation of the differentiation marker *Fgf5*. Results were normalised with fresh (day 0) mESCs.

*Oct3/4*, *Sox2* and *Nanog* were expressed at levels similar to the initial population indicating that mESCs in the perfusion cultures remained pluripotent (Figure 7). *Rex1* expression levels remained fairly constant throughout whereas *Dppa3* levels increased by day 4. In contrast, *Fgf5* was down-regulated to approximately half its initial expression by day 4. The high expression level of the ICM marker (*Dppa3*) and the lower expression of the PrEct gene, *Fgf5*, imply that the majority of the mESC population consisted of ‘naïve’ cells, in agreement to model predictions. The model was able to capture gene expression trends for *Rex1* and *Fgf5* well, although a discrepancy in *Dppa3* expression levels was observed. *Dppa3 (Stella)* expression is highly sensitive to culture conditions given the drastic changes observed in the *Stella^+^* cell population when mouse embryonic fibroblast (MEF) feeder layers and histone deacetylase inhibition are used [27]. This high degree of *Dppa3* responsiveness to culture conditions suggests that the metabolic stress gene regulatory mechanism utilized in the model cannot fully account for all the observed patterns of gene expression. Our study demonstrates how batch cultures are susceptible to stress from metabolite accumulation, which exceeds toxic levels leading to the loss of pluripotency over time. In contrast, perfusion cultures maintain relatively low levels of metabolic stress, which facilitates the maintenance of high pluripotency levels. The main findings from this study are summarized in Figure 8. The lack of detailed gene regulation networks represents the model’s weakness in predictive capability. Nevertheless, this is the first attempt, to our knowledge, of a mathematical model that accounts for culture conditions to determine the gene-regulated extent of differentiation in ESC populations.

## Discussion

Herein, it has been hypothesized that accumulated metabolic toxicity due to the mode of bioprocess operation, even in the presence of sufficient growth factors, results in loss of ESC pluripotency. A combined experimental-modeling approach was employed to account for the complexity of the system and the diverse nature of the measured variables (cell number, nutrient/metabolite concentrations, gene expression, growth factor availability) [43] in an attempt organize and maximize the amount of information extracted from experiments. Herein, a unique combination of a novel perfusion bioreactor and an attendant multi-scale mathematical model was used to establish the deficiencies in batch–type ESC cultures routinely used for production. It was demonstrated that the perfusion system effectively regulates metabolite accumulation and the model successfully predicted the performance of both perfusion and batch cultures. This integration of experimentation with mathematical modeling facilitates the optimization of ESC bioprocessing. However, further testing of significant bioprocess parameters affecting pluripotency, such as perfusion rate, need to be investigated in order to establish the wider applicability of the model.

Batch operational mode results in metabolic stress that affects mESC growth and quality (pluripotency). The mathematical model developed linked the quality by incorporating information from pluripotency-associated genes to the metabolic state of the culture. The model suggested a possible mechanism by which the accumulation of metabolites at toxic levels, even though growth factor (LIF) availability was not limited, favored the proliferation of the ‘primed’ mESC population (*X_D_*). In reality, the cultured mESCs exist in more than the two extreme states considered by the model. This however, is the first attempt to our knowledge to predict the quality of ESCs blending information from the genetic level with the metabolic status of the culture. In contrast, the perfusion operational mode alleviated the accumulation of toxic metabolites and supported higher cell density throughout the culture period, in agreement with others [41]. The model predicted that high quality (‘naïve’) mESCs preferentially expanded compared to ‘primed’ cells throughout the culture period. The modeling results were supported by the experimental data (population averaged gene expression levels and supporting flow cytometry analysis). Indicatively, the differentiation-related *Fgf5* gene was down-regulated in the perfusion cultures whereas it was up-regulated in the batch cultures. LIF availability was at comparable levels with batch cultures which suggests that LIF availability was unable to prevent the transition of ESCs from a ‘naïve’ to the ‘primed’ state seen in batch cultures. Use of population phenotyping analysis (e.g. flow cytometry) along with gene expression analysis provides further insight into ESC interaction with secreted metabolites. Specifically, the higher levels of gene expression for different lineages in batch compared to perfusion cultures reveal that metabolite stress (even with sufficient LIF) resulted in differentiation towards the mesoderm and trophectoderm lineages but a limited extent towards the neuroectoderm (Figure S3). In addition, the combination of *CD9* and *PECAM1* flow cytometric analysis enables the distinction between ‘naive’ and lineage ‘primed’ mESC populations. Both batch and perfusion cultures showed similar levels of *CD9*– indicating that the mESCs under batch conditions were not fully differentiated. However, *PECAM1+* cells were reduced in batch culture conditions compared to perfusion (Figure S3).

Due to the different metabolic characteristics of perfusion culture, the model had to be re-adjusted in order to capture the experimental results. The criterion to partition into significant and insignificant parameters was assisted through the usage of GSA. Interestingly, the parameters deemed as significant and re-estimated for perfusion indicated higher nutrient-cell growth yields, implying that perfusion culture conditions support greater levels of bioprocess productivity. Additionally, the re-estimated parameters also point towards a prioritization of glutamine utilization in perfusion cultures as opposed to glucose in batch cultures. Increased rates of glutaminolysis are apparent in *cMYC* (a pluripotency promoting factor) transformed cells [49], which further indicate that perfusion feeding is ideal for the expansion of undifferentiated ESCs. However, widespread applicability of the multi-scale model has not been fully established herein. Additional work is required to demonstrate the model’s ability to predict ESC behavior under different bioprocess parameters (e.g. perfusion rate, nutrient inoculate etc.) before applying it to globally optimize ESC cultures.

Understanding and regulating metabolism is essential for optimal ESC bioprocess design. Even though the availability of LIF/FCS was abundant, the metabolic stress experienced by the mESCs in the batch cultures was sufficient to result in loss of pluripotency. Comparison between batch, fed-batch (daily medium exchange) and perfusion cultures revealed that metabolic stress-mediated spontaneous differentiation is dependent on exposure time instead of absolute values. In fed-batch conditions, daily media exchange facilitated the clearance of toxic metabolites due to decreased exposure time (Figure S4). Previous work demonstrated lactate inhibition following a 72 hour exposure [10]. Nonetheless, glucose consumption and lactate production were higher in the fed-batch compared to the perfusion cultures. Currently, the importance of metabolism on ESC state is being recognized by recent studies on the critical role of amino acids in ESC maintenance [50] and development [51], the application of metabolomics to facilitate the dissection of metabolic pathways [52], as well as the role of metabolism in induced pluripotent stem cell reprogramming [53], [54], where the intimate link between metabolism and ‘stemness’ has been illustrated by the correlation between metabolism and reprogramming efficiencies [54]. The ability of growth factors to exert their morphogenetic influence also appears to be contextually dependent [55].

Clinical translation of pluripotent cell-related therapies depends on the development of robust, efficient and reproducible bioprocesses that will deliver the quantity and quality of the required cellular product. Recent developments on production of ‘patient-specific’ human induced pluripotent stem cells in bioreactor cultures to improve scalability [56] as well as their *in situ* reprogramming in scalable culture platforms [57], [58] further signify the importance of bioprocessing. Herein, we present a combined experimental-modeling platform for the bioprocessing of ESCs that facilitates efficient *in silico* identification of optimal culture protocols that are implemented in a novel, scalable perfusion bioreactor that provides a robust and controlled metabolic environment that is conducive to high cell growth and quality ESC bioprocessing.

## Supporting Information

Figure S1 **Primer sequences used in this article.**
(DOC)

Figure S2 **Global sensitivity analysis and results of bioprocess model parameters.**
(DOC)

Figure S3 **Further pluripotency analysis of Batch and Perfusion cultures.** A) Lineage marker (*Sox1*, *Cdx2*, *Gata4*, *Sox17*, *Goosecoid*, *Fgf5*) gene expression levels to gauge extent of spontaneous differentiation in Batch and Perfusion culture. B) Flow cytometry to assess proportion of *CD9+* and *PECAM1+* population in Batch and Perfusion culture. C) Representative dot-plot images of *CD9+* and *PECAM1+* cell populations in Batch (i) and Perfusion (ii) culture.
(DOCX)

Figure S4 **Comparison of growth, pluripotency and metabolism for Batch, Fed-batch and Perfusion.** a) Growth kinetics of Batch, Perfusion and Fed-batch cultures. b) Gauging LIF signalling activity using the expression of *Socs3*, *Stat3* and *Klf4* gene expression. Metabolic activity of Batch, Perfusion and Fed-batch cultures: glucose (c) and lactate (d). Gene expression levels of: e) *Fgf5* and f) *Dppa3* as representative differentiation and pluripotency markers respectively.
(DOCX)
