# Supplementary material for: Improving Embryonic Stem Cell Expansion through the Combination of Perfusion and Bioprocess Model Design
Source: PLoS One. 2013 Dec 10;8(12):e81728. doi: 10.1371/journal.pone.0081728 (PMC3858261; doi:10.1371/journal.pone.0081728)
Supplement: Figure S1 — Primer sequences used in this article. (DOC) [file pone.0081728.s001.doc]

| **Gene** | **Primer Sequence** | **Annealing**  **Temperature/ °C** |
| --- | --- | --- |
| Oct3/4 (S) | GGCTTCAGACTTCGCCTCC | 58 |
| Oct3/4 (A) | AACCTGAGGTCCACAGTATGC |  |
| Sox2 (S) | GCGGAGTGGAAACTTTTGTCC | 58 |
| Sox2 (A) | CGGGAAGCGTGTACTTATCCTT |  |
| Nanog (S) | CCTGATTCTTCTACCAGTCCCA | 58 |
| Nanog (A) | GGCCTGAGAGAACACAGTCC |  |
| Rex1 (S) | TGGAAGCGAGTTCCCTTCTC | 58 |
| Rex1 (A) | GCCGCCTGCAAGTAATGAG |  |
| Dppa3 (S) | GACCCAATGAAGGACCCTGAA | 58 |
| Dppa3 (A) | GCTTGACACCGGGGTTTAG |  |
| Fgf5 (S) | TGTGTCTCAGGGGATTGTAGG | 58 |
| Fgf5 (A) | AGCTGTTTTCTTGGAATCTCTCC |  |
| GapDH (S) | CATCACCATCTTCCAGGAGC | 58 |
| GapDH (A) | ATGCCAGTGAGCTTCCCGTC |  |
| Stat3 (S) | AGCTGGACACACGCTACCT | 60 |
| Stat3 (A) | AGGAATCGGCTATATTGCTGGT |  |
| Socs3 (S) | ATGGTCACCCACAGCAAGTTT | 60 |
| Socs3 (A) | TCCAGTAGAATCCGCTCTCCT |  |
| Klf4 (S) | CCAGCAAGTCAGCTTGTGAA | 60 |
| Klf4 (A) | GGGCATGTTCAAGTTGGATT |  |
| Sox1 (S) | GCACACAGCGTTTTCTCGG | 60 |
| Sox1 (A) | ACATCCGACTCCTCTTCCC |  |
| Id1 (S) | CCTAGCTGTTCGCTGAAGGC | 60 |
| Id1 (A) | GTAGAGCAGGACGTTCACCT |  |
| Id3 (S) | CTGTCGGAACGTAGCCTGG | 60 |
| Id3 (A) | GTGGTTCATGTCGTCCAAGAG |  |
