# Supplementary material for: Improving Embryonic Stem Cell Expansion through the Combination of Perfusion and Bioprocess Model Design
Source: PLoS One. 2013 Dec 10;8(12):e81728. doi: 10.1371/journal.pone.0081728 (PMC3858261; doi:10.1371/journal.pone.0081728)
Supplement: Figure S2 — Global sensitivity analysis and results of bioprocess model parameters. (DOC) [file pone.0081728.s002.doc]

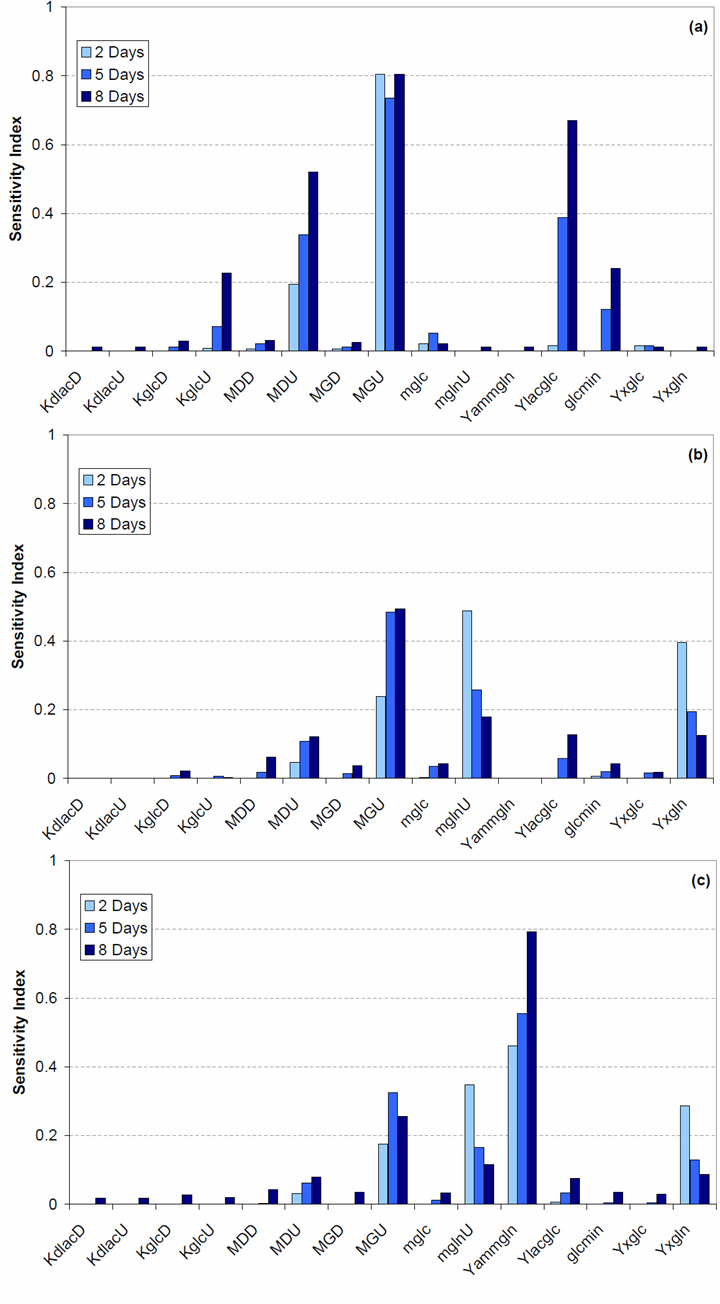


Figure S3: GSA results and methodology

SA allocates model uncertainty to the various sources of uncertainty (i.e. model parameters) facilitating the targeted reduction of output uncertainty by accurate parameter estimation through tailor made experiments. On the other hand parameters indicated as insignificant, with respect to the model output, can be fixed at their literature values (if available) or approximated. the benefits of using global sensitivity analysis (GSA) methods were illustrated when studying non-linear systems [1]. In a nutshell, the ability to estimate higher level indices, which quantify the effect on the output of parameter-parameter interactions gives global methods the edge over their local counterparts.

The highly non-linear nature of models of biological systems favours the use of global methods. Herein we have utilised the Sobol Global Sensitivy indices [2] as the GSA method of choice due to their ability to distinguish between first order sensitivity and non-liner effects. As a result the Sobol’ sensitivity indices can be used to exclude parameters with a high level of non-linear interactions from DOE on the basis of singularity. The model was simulated for 8 d of batch culture time and SA was performed at three characteristic time points (2, 5 and 8 d). Sensitivity Indices (SI’s) change dynamically along the time trajectory of the model output. As the culture progresses and nutrients start being depleted, the model output will become more sensitive towards parameters affecting nutrient uptake and metabolism. SA was conducted at different phases of the cell culture in order to capture the dynamics of the various phases of a batch culture. Specifically time points from the lag, exponential growth and decline phases were evaluated. The simulations involved scanning of all model parameters with respect to the output variables of interest. The uncertainty range associated with the parameters was set to ± 90% from the nominal value. Changes in the value of the SIs can be observed as the culture progresses.

**References**

1. Chan, K., Saltelli, A., Tarantola, S. (1997). Sensitivity Analysis of the model output: variance-based methods make the difference. Proceedings of the 1997 Winter Simulation Conference
2. Sobol', I. M. (2001). Global sensitivity idices for nonlinear mathematical models and their Monte Carlo estimates, Math. and Comp. in Simulation 55, 271-280
