# Supplementary figures and images for: Improving Embryonic Stem Cell Expansion through the Combination of Perfusion and Bioprocess Model Design
Source: PLoS One. 2013 Dec 10;8(12):e81728. doi: 10.1371/journal.pone.0081728 (PMC3858261; doi:10.1371/journal.pone.0081728)

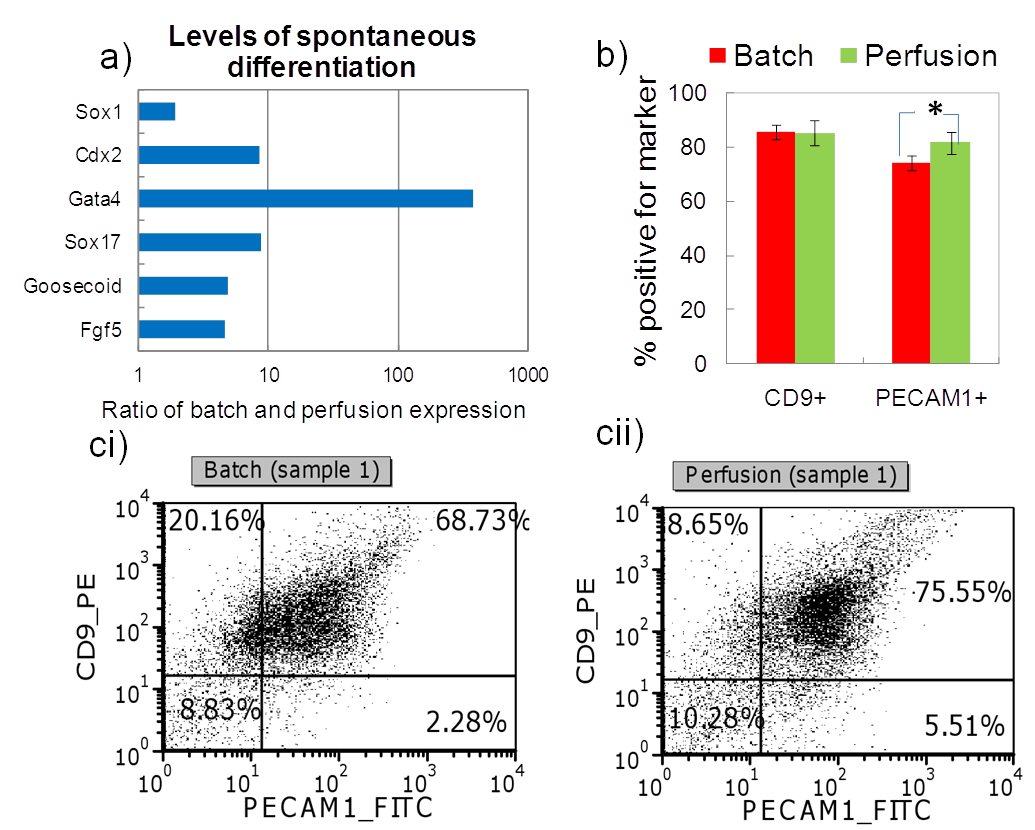


Supplemental Figure S3: Further pluripotency analyses of batch and perfusion cultures on day 6

Supplement: Figure S3 — Further pluripotency analysis of Batch and Perfusion cultures. A) Lineage marker (Sox1, Cdx2, Gata4, Sox17, Goosecoid, Fgf5) gene expression levels to gauge extent of spontaneous differentiation in Batch and Perfusion culture. B) Flow cytometry to assess proportion of CD9+ and PECAM1+ population in Batch and Perfusion culture. C) Representative dot-plot images of CD9+ and PECAM1+ cell populations in Batch (i) and Perfusion (ii) culture. (DOCX) [file pone.0081728.s003.docx]

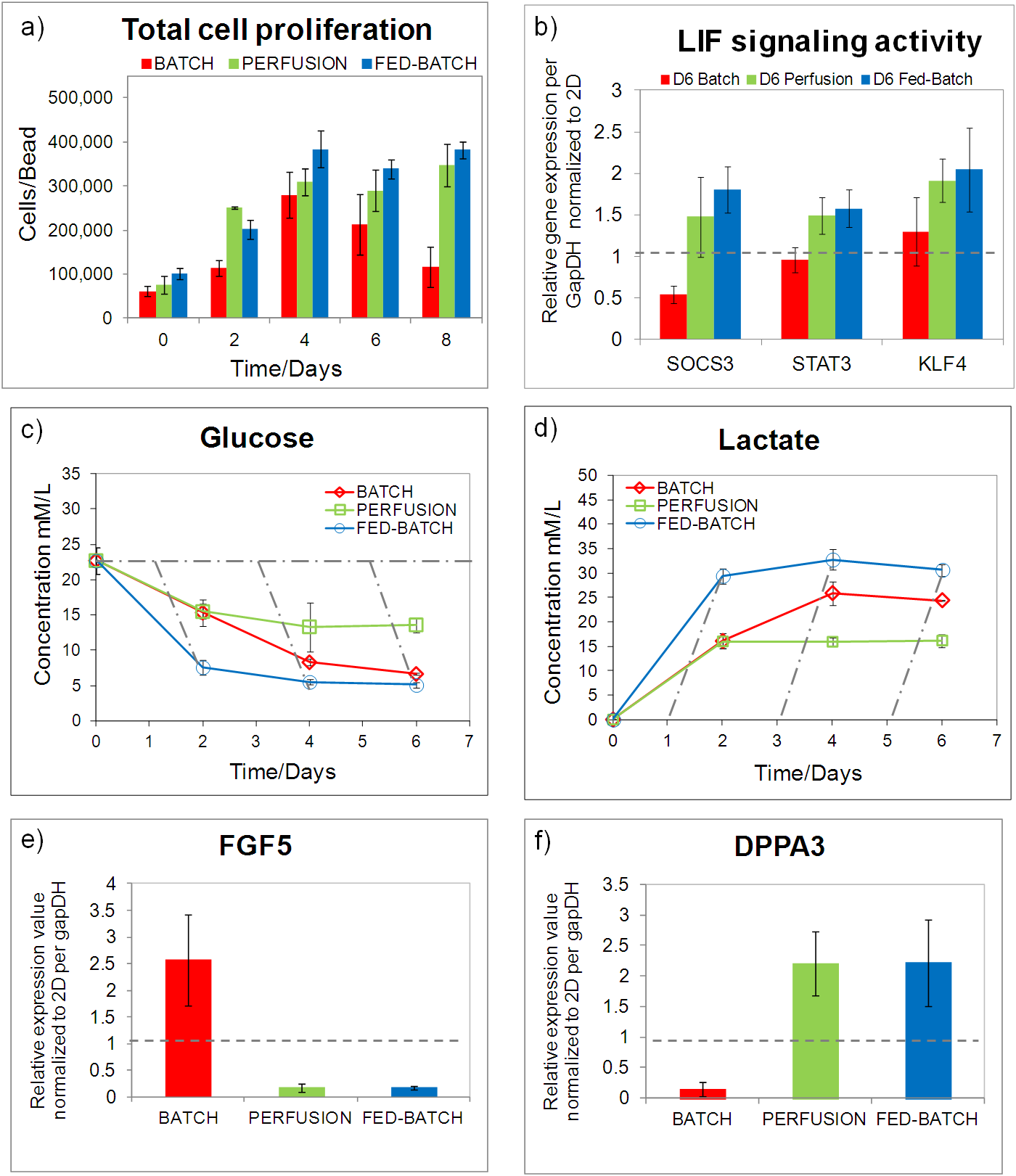


Supplemental Figure S4: Comparison between batch, fed-batch and perfusion 3D mESC cultures

Supplement: Figure S4 — Comparison of growth, pluripotency and metabolism for Batch, Fed-batch and Perfusion. a) Growth kinetics of Batch, Perfusion and Fed-batch cultures. b) Gauging LIF signalling activity using the expression of Socs3, Stat3 and Klf4 gene expression. Metabolic activity of Batch, Perfusion and Fed-batch cultures: glucose (c) and lactate (d). Gene expression levels of: e) Fgf5 and f) Dppa3 as representative differentiation and pluripotency markers respectively. (DOCX) [file pone.0081728.s004.docx]
